# Supplementary material for: External morphology of eyes and Nebenaugen of caridean decapods–ecological and systematic considerations
Source: PeerJ. 2015 Aug 18;3:e1176. doi: 10.7717/peerj.1176 (PMC4548503; doi:10.7717/peerj.1176)
Supplement: Supplemental Information 2 [file peerj-03-1176-s002.docx]

Nebenaugen dataset metadata

| Heading | Explanation |
| --- | --- |
| Family | Shrimp family as per De Grave and Fransen (2011) but considering Acanthephyridae and Oplophoridae as a single family (Wong et al., 2014) |
| Genus | Genus |
| species | species |
| Eye | Eye type as described in Figure 2 (NA = no eye) |
| Water | Marine or freshwater species |
| Rostrum | Length of rostrum |
| rostc | Rostrum class: Small (0-0.49), Medium (0.5-0.99), large (>0.99) as a proportion of carapace length. |
| depth | Depth at which specimen was found |
| depthc | Depth class; Coastal 0-100 m, Shelf 101-300 m, Deep, > 300 m |
| commensal | Living habit |
| ds | Presence or absence of a dorsal spot |
| cl | Carapace length |
| ed | Eye diameter |
| dsd | Nebenauge diameter |
| dvm | Diurnal vertical migrator or not |
| paper | Were the data taken from a source paper or specimen |
