# Supplementary figures and images for: External morphology of eyes and Nebenaugen of caridean decapods–ecological and systematic considerations
Source: PeerJ. 2015 Aug 18;3:e1176. doi: 10.7717/peerj.1176 (PMC4548503; doi:10.7717/peerj.1176)

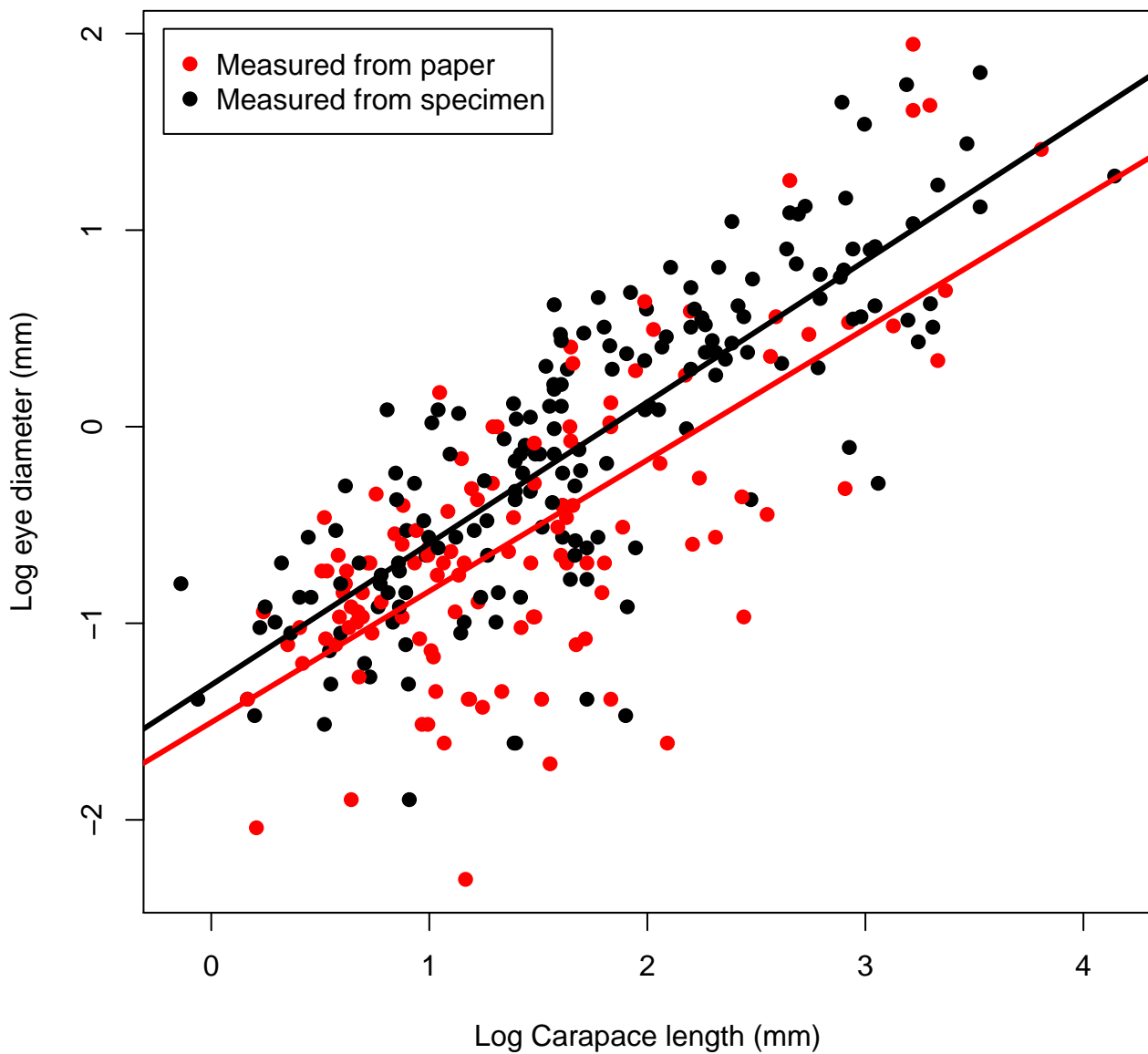

Supplement: Figure S1 — Whether or not measurements were taken from paper or physical samples has a significant effect was tested using linear regression and whether the measurement was taken from a specimen or paper as a factor. There was a significant relationship betweenlog eye diameter and carapace length (Ed = 0.79 ∗ Cl − 1.28, r2 = 0.623, F = 241.9, d.f. = 2, 290, p < .0001) but the source of the measurement was found to be significant (p < 0.0001). The decision was taken therefore to use only measurements from physical specimens for analyses. [file peerj-03-1176-s003.pdf]

Relative eye diameter

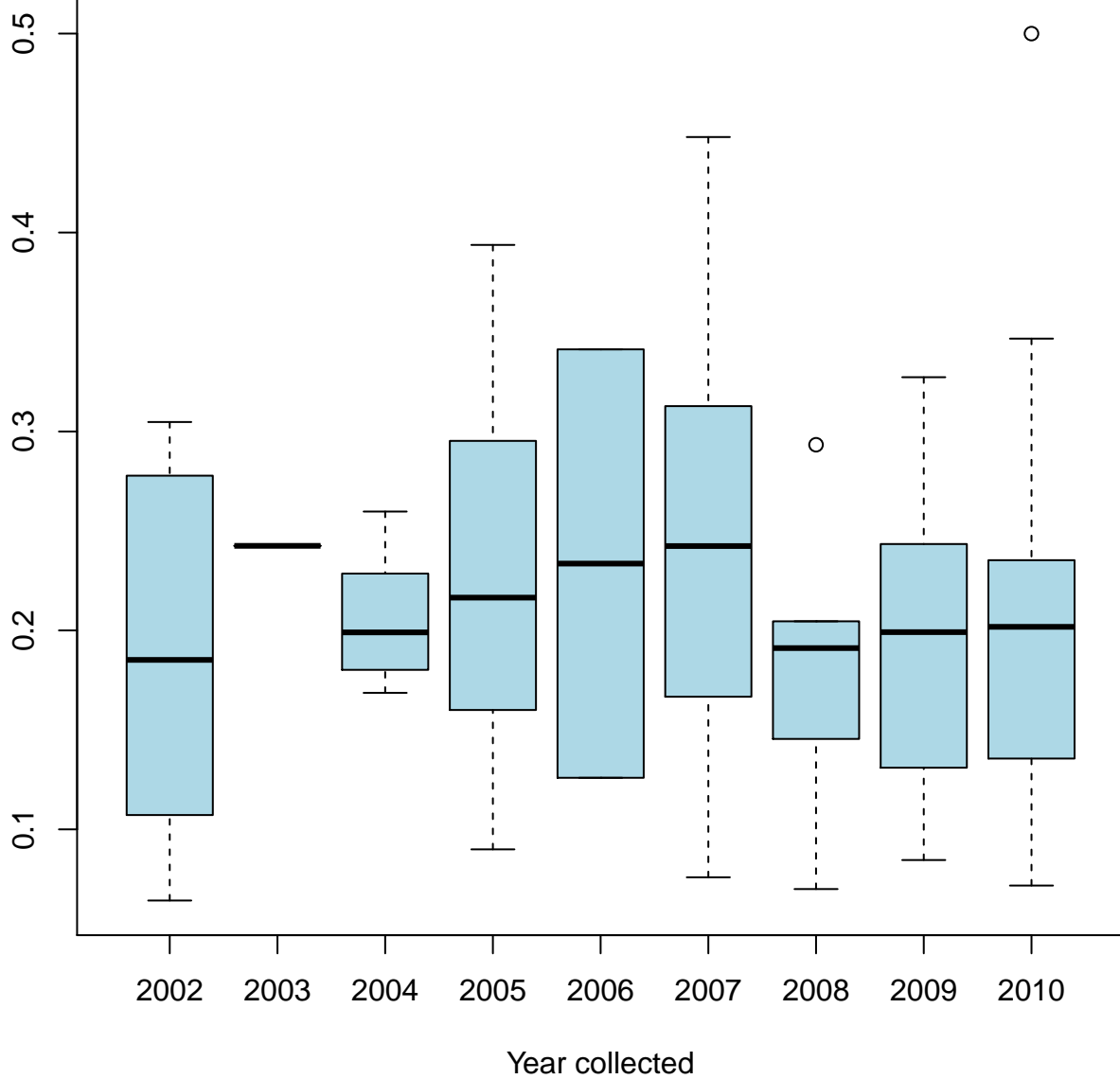

Supplement: Figure S2 — Relative eye diameter (eye diameter/carapace length) of decapod specimens collected between 2002 and 2010. No pattern is evident (Kruskal-Wallace = 3.582, p-value = 0.8927) that suggests we should not use animals from collections because of possible shrinkage. [file peerj-03-1176-s004.pdf]
